# Inorganic-Organic Hybrid Nanomaterials for Therapeutic and Diagnostic Imaging Applications

**DOI:** 10.3390/ijms12063888

**Published:** 2011-06-10

**Authors:** Juan L. Vivero-Escoto, Yu-Tzu Huang

**Affiliations:** 1 Department of Chemistry, University of North Carolina at Chapel Hill, Chapel Hill, NC 27599, USA; 2 Department of Bioenvironmental Engineering, Chung Yuan Christian University, Chung Li, 32023, Taiwan; E-Mail: yt_huang@cycu.edu.tw

**Keywords:** hybrid nanoparticles, therapeutic and diagnostic imaging applications, nanomedicine

## Abstract

Nanotechnology offers outstanding potential for future biomedical applications. In particular, due to their unique characteristics, hybrid nanomaterials have recently been investigated as promising platforms for imaging and therapeutic applications. This class of nanoparticles can not only retain valuable features of both inorganic and organic moieties, but also provides the ability to systematically modify the properties of the hybrid material through the combination of functional elements. Moreover, the conjugation of targeting moieties on the surface of these nanomaterials gives them specific targeted imaging and therapeutic properties. In this review, we summarize the recent reports in the synthesis of hybrid nanomaterials and their applications in biomedical areas. Their applications as imaging and therapeutic agents *in vivo* will be highlighted.

## 1. Introduction

Nanotechnology is a multidisciplinary and interdisciplinary scientific area and covers fields including materials science, chemistry, biology, physics, engineering and biomedicine. Nanotechnology makes use of the novel chemical and physical properties of nanoscale (1–1000 nm) materials that cannot be achieved by their bulk counterparts. For example; inorganic nanoparticles such as quantum dots (QDs) are nanomaterials generally composed of elements from either group I–VII, II–VI or III–V. QDs are nearly spherical semiconductor particles with diameters in the order of 2–10 nm, containing roughly 200–10,000 atoms. QDs exhibit luminescent properties with a controllable wavelength ranging from the visible to near infrared (NIR) according to their size [1]. Gold nanoparticles (AuNPs) have been synthesized with controllable morphology and exhibit unique surface plasmon resonance (SPR) properties. These features have been used to engineer AuNPs with strong absorption in the NIR region [2]. Moreover, AuNPs have also been explored for photothermal therapy; the heat generated through the absorbed light by small AuNPs results on hyperthermia that have been used to decrease cell proliferation [3,4]. Another class of inorganic nanoparticles is magnetite (Fe_3_O_4_) nanomaterials that are superparamagnetic and exhibit high magnetization in the presence of an external magnetic field; however, no residual magnetization is observed in its absence [5]. For instance, these magnetite-based nanoparticles have been used for magnetic resonance imaging (MRI) contrast agents [6]. In the case of silica-based nanoparticles; two major types have been widely explored, solid silica nanoparticles (SNPs) and mesoporous silica nanoparticles (MSNs). In contrast to the previously described inorganic nanomaterials, SNPs do not acquire any peculiar property from their sub-micrometric size, except for the corresponding increase of surface area [7]. What makes SNPs very exciting from a nanomedicine point of view is the presence of a well-defined structure (size, surface chemistry, morphology, porosity, shells, *etc.*) that can be easily engineered with the desired properties and functionalized or doped with organic/inorganic species [8]. Unlike SNPs, MSNs exhibit many outstanding properties such as high surface areas, tunable pore sizes, and large pore volumes [9,10]. All these inorganic nanoparticles can be further functionalized with organic moieties, through different synthetic strategies, to afford relevant nanomaterials for biomedical applications. For instance, therapeutic and diagnostic imaging agents can be attached to the surface of inorganic nanoparticles. In addition, the external surface can be passivated with polymers, proteins, and carbohydrates to endow the material with specific biological properties. Also these nanomaterials can be functionalized with targeting groups such as receptor ligands (folic acid), anti-bodies, peptides, aptamers, DNA, *etc.* to afford target specific vehicles that have an important application in drug delivery for cancer treatment. At the end, these hybrid nanoparticles composed of both inorganic nanoparticles and organic moieties will not only retain the beneficial features of both inorganic and organic fragments, but also possess unique advantages for imaging and therapeutic applications. In this review, we will explore the wide variety of synthetic strategies for the functionalization of inorganic nanoparticles with organic molecules and macromolecules. Moreover, the application of these hybrids platforms in therapy and diagnostic imaging will be described. Finally, a special attention will be devoted to theranostic systems and *in vivo* applications.

## 2. Solid Silica Nanoparticles (SNPs)

Recently, SNPs have attracted a great deal of attention due to their chemical and physical versatility, and biocompatibility. In addition, silica nanoparticles are highly hydrophilic and easy to centrifuge for separation, surface modification, and labeling procedures. This silica-based materials exhibit enhanced and controllable mechanical and chemical stability and their porosity can also be easily tailored.

### 2.1. Synthesis

The SNPs are generally synthesized using two major strategies: sol-gel synthesis and microemulsion synthesis. The first method, developed by Stöber and coworkers in the late 1960s [11], involves the controlled hydrolysis and condensation of a silica precursor, such as tetraethoxysilane (TEOS), in ethanol solution containing water and ammonia as a catalyst. The size of the particles can be tuned by adjusting the reaction conditions [12]. The nanoparticles obtained by this method are fairly monodisperse silica particles with diameters ranging from 30 nm to 2 μm. Moreover, these nanoparticles remain stable in solution due to electrostatic repulsion from their negative surface charges. The second synthetic approach was developed by Arriagada and Osseo-Asare in the early 1990s and involves the ammonia-catalyzed polymerization of TEOS in a reverse phase, or water-in-oil, microemulsions [13,14]. Reverse phase microemulsions are highly tunable systems that consist of nanometer-sized water droplets stabilized by a surfactant in the organic phase. The single-phase microemulsion system is both isotropic and thermodynamically stable. The micelles of the microemulsion act as “nanoreactors” where the particle growth occurs and the final size is controlled by the water/organic solvent ratio [15]. With this method highly monodisperse and perfectly spherical particles are obtained with sizes ranging from 20 to 100 nm. In that sense, the reverse microemulsion method is superior to the Stöber method for producing monodisperse silica nanoparticles smaller than 100 nm.

SNPs can be functionalized by the addition of hydrophilic functional molecules; which allows incorporation of organic species in the silica matrix. In this case, functional molecules are entrapped in the silica framework via noncovalent interactions; for example, fluorophores such as the positively charged Ru(bpy)_3_^2+^ can be doped in the nanoparticles in order to develop fluorescent nanoparticles [16]. Interestingly, entrapped fluorophores exhibit a higher quantum yield and stronger photostability than the free molecules. Functional molecules can also be integrated through organoalkoxysilanes derivatives [17]. In this way, the molecules are chemically incorporated within the silica matrix via silanol linkages, leading to stable hybrid silica nanoparticles with uniform agents throughout the nanoparticle that are protected from the environment. In addition, surface functionalization can also be achieved by reacting preformed silica nanoparticles with trialkoxysilane derivatives [18]. Post-synthetic grafting is particularly useful for modifying the particle surface with selected agents that are not stable during the silica particle synthesis. Finally, the ability to synthesize nanoparticles with core-shell architectures allows multiple functions to be brought together within a single vehicle, separated as different shells [19].

### 2.2. Therapeutic Applications

SNPs are promising candidates for improved drug delivery systems. Drug molecules can be loaded into SNPs, and surface modification of the nanoparticles with targeting groups allow specific cells or receptors in the body to be localized [17,20]. Upon target recognition, NPs can release the therapeutic agents at a rate that can be precisely controlled by tailoring the internal structure of the material for a desired release profile. For instance, Prasad and coworkers described the use of SNPs for photodynamic therapy (PDT) [21]. A known photosensitizer and a two-photon energy donor were co-encapsulated in a 30 nm SNP. Upon two-photon irradiation, the photosensitizer is indirectly excited through fluorescence resonance energy transfer (FRET), resulting in the generation of singlet oxygen. The uptake and therapeutic effect of these particles were demonstrated through fluorescence imaging of HeLa cells. Hai and coworkers reported a similar approach for the synthesis of 105 nm SNPs with entrapped methylene blue (MB) dyes for near-IR (NIR) imaging and PDT [22]. The therapeutic effect of this platform was demonstrated *in vitro* by using HeLa cells. Significant toxicity was only observed in cells treated with the MB nanoparticles and laser irradiation. Both fluorescence imaging and PDT was observed *in vivo* in a mouse xenograft model. The nanoparticles were injected directly in the tumor, after laser treatment, the tumor become necrotic.

SNPs are also promising materials as DNA carriers for gene therapy [17,23,24]. Recently, the potential of cationic SNPs was investigated for *in vivo* gene transfer [25]. These particles were evaluated for their ability to transfer genes in a mouse lung. Two-fold increase in the expression levels was found with silica particles in comparison to enhanced green fluorescent protein (EGFP) alone. In addition, Prasad and coworkers have developed a fluorescently labeled SNPs with cationic surface coating [26]. This system was tested as DNA carrier in *in vitro* and *in vivo* conditions. Confocal microscopy studies revealed that the nanoparticles were uptaken by cells and the released DNA migrating toward the nucleus. Moreover, *in vivo* studies showed that the particles were able to successfully transfect and modulate the activity or neural cells in a murine model.

### 2.3. Diagnostic Imaging Applications

SNPs have been extensively studied as luminescent material for a wide variety of applications in biotechnology and medicine [20,27]. Cancer cell imaging has been one of the major areas of research and different strategies have been explored for using SNP probes to target cancer cells. For instance, primary or secondary antibodies have been covalently immobilized onto the SNP surface in order to selectively and efficiently bind various cancer cells [17]. In addition, receptor ligands and recognition peptides can also be attached onto SNPs in order to label cell-membrane proteins. In this way, folic acid and TAT have been utilized to target SCC-9 and human lung adenocarcinoma (A549) cells. Recently, aptamers have also been used as a novel class of ligands. Aptamers are short strands of DNA/RNA for recognition of a variety of targets including proteins and small molecules as well as complex samples. Specific targeting and visualization of acute leukemia cells with aptamer-conjugated SNPs have been developed using laser scanning confocal microscopy (LSCM) and flow cytometry [28]. In addition, fluorescent SNPs have been exploited as probes for DNA/microarray detection. The first lab-based trial was based on s sandwich assay [29]. Single nucleotide polymorphism detection is also feasible by developing Cy3- and Cy5-doped Au/silica core-shell nanoparticles. The NP-based DNA detection strategy can be extended to the use of SNPs as fluorescent labels for DNA and protein microarray technology in order to meet the critical demand for enhanced sensitivity [30].

**Figure 1:**
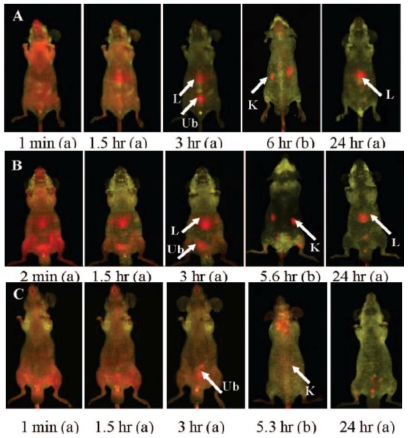
*In vivo* imaging biodistribution of different i.v. injected surface-modified SiNPs at different time points, postinjection (**A**–**C**; (a), abdomen imaging; (b), back imaging): **(A)** OH-SiNPs; **(B)** COOH-SiNPs; **(C)** PEG-SiNPs. Arrows mark the location of the kidney (K), liver (L), and urinary bladder (Ub). Reproduced with permission from [31]^®^ 2008, American Chemical Society.

**Figure 2:**
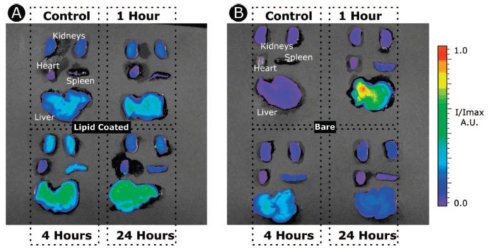
Fluorescence imaging of liver, spleen, kidneys, and heart of control mice and mice sacrificed 1, 4, and 24 h post-injection with **(A)** Lipid-coated SiNPs and **(B)** Bare-SiNPs. While an immediate uptake of bare silica particles in the liver was observed, the lipid-coated silica particles accumulated gradually over time in the liver which is in agreement with their prolonged circulation half-life value. Reproduced with permission from [32]^®^ 2008, American Chemical Society.

*In vivo* imaging applications of SNPs have been addressed to study the biodistribution and pharmacokinetics of this material. For example, the study of the biodistribution in real time of SNPs was first reported by K. Wang and coworkers using Ru(byp)_3_^2+^-doped SNPs with different surface coatings (OH, COOH, and monomethyl ether PEG (MW ~ 428)) on nude mice by optical imaging (ex: 465–495 nm; em: 515 nm long-pass) [31]. The authors found that the blood circulation time and clearance half-life are surface coated dependent, PEG-, OH-, and COOH-SNPs exhibited blood circulation life time (t_1/2_) of 180 ± 40 min, 80 ± 30 min and 35 ± 10 min, respectively. The SNPs were located mainly in the liver, urinary bladder, and kidney in a time dependent manner. Interestingly, the *in vivo* optical imaging results showed that independently of the surface chemistry, SNPs were presented in some organs involved in the formation and excretion of urine, as an indication that part of the SNPs are cleared through the renal route (Figure 1). Other strategies to functionalize the surface of SNPs have been explored; for instance, the use of phospholipids for coating inorganic nanoparticles is well-established and has been one of the most successful strategies of nanotechnology for biomedical applications. QD containing SNPs of ~35 nm in diameter were coated with both a monolayer of PEGylated phospholipids (PEG(2K)-DSPE) and a paramagnetic lipid coating (Gd-DTPA-DSA) [32]. The short-term cytotoxicity and PK of this platform was investigated by fluorescence imaging, MRI, ICP-MS, LSCM and TEM. This wide variety of complementary techniques allowed investigating the performance of the lipid-coated QD-SNPs material at different levels; from organ, tissue, cellular, and at subcellular level. The PEG-lipid coating increased the blood circulation time by a factor of 10; from 14 ± 2 min for the bare SNPs to 162 ± 34 min. The bare SNPs accumulate in the liver, spleen, and lungs; however, SNPs were not observed in kidneys. In the case of lipid-coated SNPs the main accumulation is in the liver and spleen; nevertheless, the accumulation rate is much slower than bare SNPs (Figure 2). The influence of particle size in the biodistribution and PK of SNPs *in vivo* has also being studied by fluorescence imaging modality. Nanoparticles containing a fluorescence group (Rhodamine B isothiocyanate–RITC) with 50, 100 and 200 nm in size were synthesized and characterized (50-, 100-, and 200-SNPs, respectively) [33]. The *in vivo* data show that 50-SNPs are excreted faster by both renal and hepatobiliary route than 100- and 200-SNPs. The fluorescence intensity of all three sized SNPs was detected in the kidney, the liver and spleen; nevertheless, the 200-SNPs are taken up faster and in a higher amount than the smaller-size particles by macrophages of the spleen and liver. This study demonstrated that tissue distribution and excretion are different depending on particle size. Multimodal SNP-based imaging probes have also been used to quantify the biodistribution of silica materials *in vivo*. Recently, Prasad and coworkers studied quantitatively the biodistribution and PK of organically modified SNPs (ORMOSIL) [34]. They synthesized a 20 nm NIR dye DY776 containing SNPs, this particle was further functionalized with PEG chains and ^124^I Bolton-Hunter reagent to afford a bimodal contrast agent with optical and positron emission tomography properties. In this work, the authors took advantage of the bimodal features of this system to quantify its biodistribution and PK by both NIR fluorescence and radioactivity measurements. The NIR images showed that DY776-SiNPs accumulate mainly in the liver and spleen (almost 75%) 2 h post intravenous injection; on the contrary, less than 5% of material was localized in the lung, kidney, and heart. This data was further corroborated by radioactivity measurements where 58 and 37% ID/g were found in the spleen and liver, respectively. This is a clear indication that these particles are taken up by macrophages in the liver and excreted with the fecal matter via the hepatobiliary transport mechanism through the stomach.

## 3. Mesoporous Silica Nanoparticles (MSNs)

**Figure 3:**
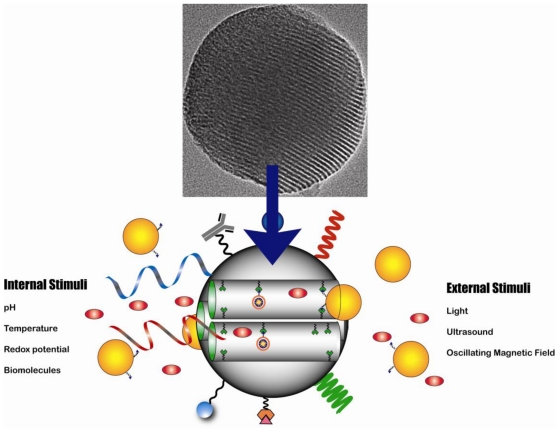
TEM image of MSNs (top), and schematic representation (bottom) of drug delivery from the same material. The release of the therapeutic agent can be triggered by different stimuli-responsive strategies (pH, redox potential, temperature, light, ultrasound and magnetic field).

MSNs have attracted a great deal of attention for their potential application in the fields of catalysis, biotechnology and nanomedicine [9,10,35–38]. MSNs are mesoporous materials, which contain hundreds of empty channels arranged in a 2D network of honeycomb-like porous structure (Figure 3). As has been described in the literature, these silica-based nanoparticles also offer several unique and outstanding structural properties, such as high surface area (>1000 m^2^/g), pore volume (>1.0 cm^3^/g), stable mesostructure, tunable pore diameter (2–10 nm), and modifiable morphology (controllable particle shape and size) [39,40]. For instance, their large surface area and pore volume allow for high loading of imaging and therapeutic agents. The tunable diffusional release of drug molecules from the highly ordered mesoporous structure gives rise to a biogenic local concentration at the targeted area, which reduces the overall dosage and prevents any acute or chronic complications. In addition, MSNs offer the ability to further functionalize the surface of MSNs with a wide variety of stimuli-responsive groups, target agents, polymers, biomolecules, molecular gatekeepers, *etc.* Finally, MSNs can effectively protect the pharmaceutical cargoes, such as drugs, imaging agents, enzymes, and oligonucleotides, from premature release and the undesired degradation in harsh environments before reaching the designated target. In summary, MSNs offer ideal characteristics to fulfill most of the requisites to develop imaging and drug delivery nanovehicles.

### 3.1. Synthesis

MSNs exhibit many unique properties such as high surface area, stable and rigid framework, tunable pore size, and large pore volume. MSNs are typically synthesized by a surfactant-templated sol-gel approach [10]. These materials possess a honeycomb-like, 2D hexagonal porous structure with hundreds of empty channels that are able to encapsulate relatively high amounts of functional molecules and shelter these moieties from exposure to the external environment. In addition; MSNs have two different surfaces, the interior pore surface and the exterior particle surface, which offer many advantages over solid nanoparticle materials. More recently, several methods to control the morphology, pore size and surface functionalization of MSNs have been developed [10,41]. These methodologies have afforded MSNs with different morphologies such as spheres, rods, twisted column and kidney-bean-shaped nanoparticles.

MSN materials can be chemically functionalized using two different approaches, post-synthetic grafting and co-condensation [35,41,42]. The former is the most popular approach for covalently incorporating organic functionalities to the mesoporous material. This method is based on a condensation reaction between a given trialkoxysilane and the surface free silanol and geminal silanol groups on the silica surface. This method allows the particle morphology and pore structure to remain intact, but it has been found that most materials functionalized via the grafting method contain an inhomogeneous surface coverage of organic functional groups. In the second approach, the desired trialkoxysilane is condensed into the pores of MSNs during the synthesis of the nanoparticles leading to homogeneous incorporation of the functional group throughout the nanoparticles [43,44]. The choice of trialkoxysilane precursors is limited to those with organic functional groups that would be soluble in water and can tolerate the extreme pH conditions that are required for the synthesis of MSNs and the subsequent removal of surfactants. The degree of functionalization, particle size, and morphology can be modified by adjusting the synthetic conditions, such as reagent concentration and the hydrophobicity/hydrophilicity of the trialkoxysilane reagents. Additionally, the combination of both synthetic methods has afforded a wide variety of multi-functional systems that have been applied in a wide variety of fields such as catalysis, biotechnology and biomedicine, just to mention some of the more prevalent representatives [38,39,45].

### 3.2. Therapeutic Applications

At the beginning of this century, the application of mesoporous silica as drug delivery vehicles was proposed by Vallet-Regi [46]. Along these years drug delivery systems based on MSNs capped with solid nanoparticles such as cadmium sulfide [47], gold [48–50], and iron oxide [51,52]; and soft nanoparticles such as dendrimers [53], proteins [54], and polymers have been developed [55–57]. This gatekeeper concept has been applied to afford site- and time-control on the release of biogenic agents based on stimuli responsive linkers (Figure 3). In addition, to achieve precise spatial and temporal delivery of therapeutic agents to target sites, a variety of stimuli-responsive groups have been introduced to MSN. These moieties respond to stimuli found internally in biological systems (pH, temperature, redox potential and biomolecules) and stimuli that can be applied externally from biological systems (light, ultrasound and oscillating magnetic field). Various responses to stimuli are feasible, including bond cleavage, competitive binding and conformational changes. MSN systems have been designed to take advantage of these responses and to trigger the release of encapsulated molecules. Several reviews addressing the application of stimuli and triggers in MSN-based drug delivery systems have already been published somewhere else [40,58,59]. Here, we will focus on the most recent applications of MSN-based drug delivery systems *in vivo*.

F. Tamanoi and coworkers published a thorough investigation on the toxicity, biodistribution, PK and therapeutic properties of MSNs [60]. The authors studied the short-term toxicity using different concentrations of MSNs (3, 6, 12.5, 25, 50 mg/Kg) for 14 days (five doses) and the long-term toxicity using a fixed concentration of 50 mg/Kg for two months (18 doses). In both experiments, no infection, impaired mobility, histological lesions nor reduced food taking was observed. The authors studied the biodistribution and excretion of MSNs by fluorescence imaging (fluorescein) and ICP-OES, respectively. The fluorescence intensity of the MSNs in tumors was much stronger than that from the other tissues at 4 and 24 h. The next strongest fluorescence intensities were found in the kidney and liver. Interestingly, after quantitatively analyzing the amount of Si excreted from the animal body the authors found out that the material is cleared through both renal and hepatobiliary pathways. The authors tested the therapeutic effects of MSNs loaded with camptothecin (CPT) as chemotherapeutic on human breast cancer cells (SK-BR-3 and MCF-7), and breast fibroblast cells (MCF10F). In addition, to enhance the tumor accumulation of MSNs, the material was further functionalized with folic acid (F-MSNs), which specifically binds to folate receptors that is up-regulated in various types of human cancers. The cytotoxicity assays demonstrated that both MSNs and F-MSNs are capable of delivering CPT into cells and exert cell-killing effects. Finally, the CPT-loaded MSNs and CPT-loaded F-MSNs were tested in nude mice with established xenografts of human breast cancer cell MCF-7. The tumors in the mice treated with these materials were virtually eliminated at the end of the experiments. These results proved that the high drug-loading ability, low toxicity, and tumor accumulating effect of MSNs provide a promising drug-delivery vehicle for anticancer drugs.

### 3.3. Diagnostic Imaging Applications

During the past few years, research in biomedical imaging has been one of the most successful interdisciplinary fields. Multimodal techniques are quickly becoming important tools for developing innovations in the areas of biomedical research, clinical diagnosis, and therapeutics [61]. For instance, tracking the biodistribution of soft tissues *in vivo* for distinguishing anatomical images and assess disease pathogenesis by biomarkers is crucial for therapeutical treatments [62]. Due to their unique properties, such as biocompatibility, optical transparency, easy incorporation of nanoparticles (*i.e.*, Au, and Fe_3_O_4_), and functionalization with optical groups (fluorescein, Rhodamine B), MSNs have attracted a great deal of attention as suitable platform for multimodal imaging and multifunctional probes.

Optical imaging has been a versatile and easy-of-use approach, in terms of availability of a variety of contrast agents for molecular targeting, avoidance of radiopharmaceuticals, and relatively low cost of instrumentation. These features make it complementary to other modalities such as MRI. The use of optical imaging agents has being prevailing for investigating cellular and intracellular imaging of MSNs [47,53]. However, for *in vivo* imaging, optical imaging usually suffers from the attenuation of photon propagation in living tissue and poor signal to noise ratio due to tissue autofluorescence. The use of NIR contrast agents is thus critical for *in vivo* optical imaging since the blood and tissues are relatively transparent in the range of 700–1000 nm wavelength so minimizing complications resulting from intrinsic background interference. Recently, L.-W. Lo and coworkers reported on the development of NIR MSN-base probes [63]. Indocyanine green (ICG) was entrapped in MSNs by electrostatic interaction. ICG is a FDA approved optical agent for clinical use; moreover, its characteristic fluorescent excitation and emission wavelengths (ex: 800 nm; em: 820 nm) in NIR window, make this agent ideal for *in vivo* imaging. Using this ICG-MSN optical imaging platform, the authors were able to noninvasively image MSN material biodistribution in both rat and mouse models. The optical images show that the nanoparticles after intravenous injection are immediately accumulated in liver followed by kidney, lung, spleen and heart. Recently, the same group reported on a systematic investigation of the effect of the surface charge of ICG-MSNs on their biodistribution [64]. The results showed that by judiciously tailoring the surface charge of MSNs it would be possible to control the MSNs rates of excretion and their biodistribution.

**Figure 4:**
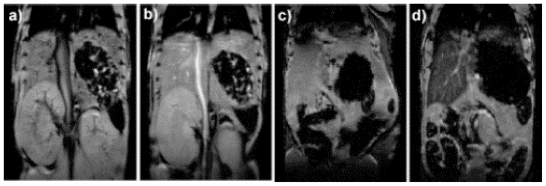
**(a)** Precontrast and **(b)** postcontrast (2.1 μmol/Kg dose) T_1_-weighted mouse MR image showing aorta signal enhancement; **(c)** Precontrast and **(d)** postcontrast (31 μmol/Kg dose) mouse MR images showing liver signal loss due to T_2_-weighted enhancement. Reproduced with permission from [68]^®^ 2008, American Chemical Society.

Among various imaging methods, MRI is currently one of the most powerful *in vivo* imaging technologies. MRI has the advantages of being a noninvasive diagnostic tool that provides high three-dimensional resolution of anatomical images of soft tissue. MRI exploits the remarkable range of physical and chemical properties of water protons (*i.e.*, hydrogen nuclei) [65,66]. In MRI the sensitivity and exceptional soft tissue contrast are further improved by the use of MR contrast agents that change the local MR signal intensity. There are two main classes of contrast agents for MRI: paramagnetic complexes and superparamagnetic iron oxide particles. The former class includes mainly chelates of Mn(II), Mn(III) and Gd(III) ions, with gadolinium-based agents being the most commonly used [67]. The MRI contrast agents currently on the market lack sensitivity and often do not provide satisfactory image contrast enhancement; because of that, high concentrations of contrast agent are required. For that reason, nanoparticulate MR contrast agents are being explored as a potential alternative. Several advantages can be envisioned by using MRI nanoprobes; for example, a high payload of a molecular contrast agent can be incorporated in a single nanoparticle, thus increasing the effective relaxivity per nanoparticle. In addition, molecular MRI contrast agents can be protected from the harsh environment under physiological conditions. Based on these advantages, MSNs have been used as a potential alternative for MRI contrast agents. Lin and co-workers demonstrated the use of MSNs as nanoparticulate T_1_-weighted MR contrast agent in *in vitro* and *in vivo* conditions [68]. The synthesis of the nanoprobe was carried out through the traditional grafting method of a silane derivative, Gd-Si-DTTA complex. The nanoparticles exhibited very large longitudinal (r_1_) and transverse relaxivities (r_2_). The material was labeled with a fluorescent agent (rhodamine B) to study the *in vitro* properties with immortalized murine monocyte cell line. Both LSCM and cell phantom images showed that the nanoparticles were successfully internalized by the monocytes. Finally, the material was intravenously injected to a mouse via tail vein to study the MR contrast enhancement properties. A T_1_-weighted contrast enhancement was clearly observed in the aorta of the mouse 15 min post-injection, this shows the potential of the Gd-MSN platform as intravascular MR contrast agent (Figure 4). Moreover, it was also demonstrated that this nanoprobe can be used as T_2_-weighted contrast agent, the authors reported the signal loss in the liver after several days of the administration of the MSN contrast agent.

D.-M. Huang, C.-Y. Mou and co-workers have explored the application of silica nanoparticles as multimodal contrast agents for tracking stem cells [69,70]. The ability to monitor cell trafficking *in vivo* and its biodistribution is a prerequisite for developing successful stem cell therapies. This research group has developed a dual-modal contrast agent platform based on MSNs. This system combines a green fluorescent agent (FITC) and a MR contrast agent. The group has reported the use of both T_2_- and T_1_-weighted MR contrast agents; small particles of iron oxide act as negative contrast agent to afford Mag-Dye@MSNs [70], and Gd-chelates were grafted to MSNs to afford the positive contrast agent Gd-Dye@MSNs [69]. Both systems were efficiently internalized into human mesenchymal stem cells (hMSCs) without affecting cell viability growth or differentiation. The efficient hMSCs tracking was visualized *in vitro* and *in vivo* by a clinical 1.5T MRI system. *In vivo*, the labeled cells remained detectable by MRI after long-term growth or differentiation, as further evidence of the biocompatibility and durability of both Mag-Dye and Gd-Dye@MSNs nanoprobes. In addition, C.-Y. Mou and coworkers used the dual-modality Mag-Dye@MSNs system to follow the biodistribution of MSNs in mice after eye vein injection [70]. The Mag-Dye@MSNs darken liver/spleen/kidneys T_2_-weighted MR images showed that the MSNs start to accumulate in these organs predominantly through a vascular mechanism in the early stages, and that the signal darkening, mainly in liver and spleen, was due to nanoparticle accumulation within the RES in the late stages. Some other approaches using DOTA as Gd-chelate have been published [71]. In addition, the use of micro-size mesoporous silica as an alternative to nanoparticulate MRI contrast agents has been recently reported [72].

### 3.4. Theranostic Applications

**Figure 5:**
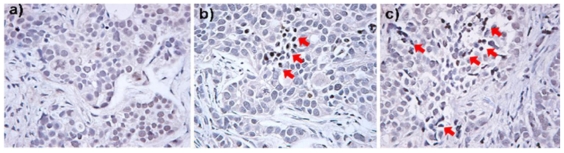
TUNEL assay for apoptotic cell death. Tumor section from mouse that was given i.v. injection of **(a)** free IO-MSN, **(b)** DOX loaded IO-MSN (DOX 2mg/Kg), and **(c)** DOX loaded IO-MSN (DOX 4 mg/Kg). Arrows indicate examples of TUNEL-positive (brown color) cells with apoptotic morphology. The mice were sacrificed 48 h after injection. Reproduced with permission from [52]^®^ 2010, American Chemical Society.

**Figure 6:**
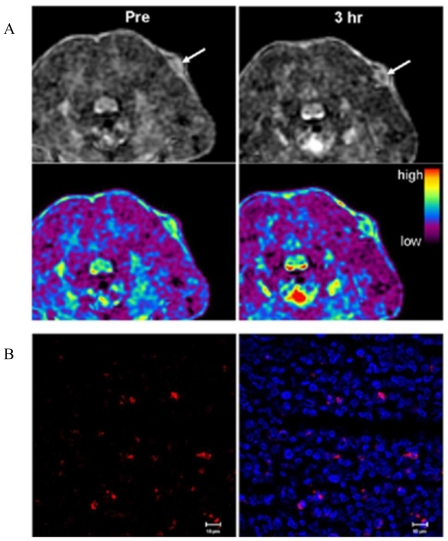
*In vivo* accumulation of iron oxide MSN at tumor site. **(A)** *In vivo* T_2_-weighted MR images (upper) and color mapped (lower) images of tumor site before and 3 h after i.v. injection of iron oxide IO-MSN (arrows indicate tumor site); **(B)** LSCM of sectioned tumor tissue harvested 24h after injection. Left; red fluorescence showing IO-MSN internalized cells. Right; Merged image with DAPI stained nuclei (blue). Reproduced with permission from [52]^®^ 2010, American Chemical Society.

The capability of developing nanoparticle-based platforms that merge both therapeutic and diagnostic properties has been the Holy Grail for nanomedicine. The first MSN-based theranostic system was reported by K. Moon, T. Hyeon and coworkers [73]. They showed the potential of MSN platform for simultaneous MR and fluorescence imaging, and for drug delivery *in vivo*. The authors synthesized a discrete and monodisperse core-shell MSNs consisting of a single iron oxide nanocrystal core and a mesoporous silica shell (IO@MSNs). The multimodal imaging capabilities of this platform were applied in MCF-7 breast cancer cells by using fluorescein and rhodamine B as imaging agents to determine the intracellular internalization of this material by LSCM. Moreover, the T_2_-weighted properties of IO@MSNs-PEG as MR contrast agent were measured; the r_1_ and r_2_ relaxivity values of the core-shell system were 3.40 and 245 mM^−1^s^−1^, respectively. To test the drug delivery properties of IO@MSNs-PEG, doxorubicin (DOX) was loaded and tested on SK-BR-3 cell line. The cytotoxicity assay demonstrated the efficacy of this nanovehicle to successfully transport and deliver DOX inside SK-BR-3 cells. The authors investigated the potential for *in vivo* imaging of IO@MSNs-PEG by tracking the passive accumulation of nanoparticles in a breast cancer xenograft model. At 2 h after intravenous injection, the accumulation of nanoparticles in tumor was detected by T_2_-weighted MR images. The accumulation of IO@MSNs-PEG was further confirmed by fluorescence imaging of tumor and several organs of sacrificed mice 24 h after injection. The same authors also published a paper on the synthesis of dye-doped iron oxide capped MSNs for multimodal imaging and drug delivery applications [52]. In this case, the fluorophore (fluorescein or rhodamine B) was doped in the interior channels and the IO nanoparticles were chemically attached on the exterior surface of MSNs. Similar to the previous study, the nanoparticles were further functionalized with PEG(5K). The T_2_-weighted properties of this material were characterized; interestingly, the assembly of multiple IO nanoparticles on MSN resulted in a remarkable enhanced MR contrast (r_2_ for free IO nanoparticles 26.8 mM^−1^s^−1^ and IO-MSN 76.2 mM^−1^s^−1^). To corroborate the *in vitro* multimodal imaging, fluorescence and T_2_ weighted MR images of IO-MSN labeled cell phantom were acquired. To examine drug delivery, the chemotherapeutic agent DOX was loaded into IO-MSNs. The antitumor efficacy of DOX loaded IO-MSN was successfully tested using the B16-F10 melanoma cell line. The platform was evaluated *in vivo* by intravenous injection into a nude mouse bearing a tumor on its shoulder. At 3 h after injection, a drop in the MR signal was detected at the tumor site, demonstrating passive targeting of IO-MSNs caused by the EPR effect. The antitumor activity was corroborated using TUNEL assay (Figure 5).The accumulation of the material on the tumor site was confirmed by fluorescence imaging on sections of the tumor tissue (Figure 6). These results showed that DOX was delivered to the tumor site successfully and its antitumor activity was retained. This report proved that MSNs can indeed deliver and release a chemotherapeutic agent to solid tumors in animals through EPR effect.

## 4. Gold Nanoparticles (AuNPs)

Metallic gold has been a fascinated material for decorative purpose since ancient times because of excellently ductile and bright properties. The modern era of gold material started since the discovery of new properties of colloidal gold by Faraday. In 1857, Michael Faraday observed that the solution of colloidal gold showed deep-red color, which was apparently different to that of gold bulk [74]. This special phenomenon was attributed to the nano-sized gold particles and the principle was well explained by Mie by solving Maxwell‘s electromagnetic equation [75,76]. Since half century ago, reliable and high-yielding methods for the synthesis of Au nanoparticles (AuNPs) have been developed [77], and AuNPs have been applied on analytical chemistry [78,79], and electronics [80]. Over past decade, AuNPs made significant process on biology and nanomedicine, especially on therapeutic and imaging applications, based on their characteristic properties such as tunable optical properties, high surface area and surface modification.

### 4.1. Synthesis

High-yielding and size-controlled AuNPs can be prepared by simple chemical synthesis to obtain nanoparticles with uniform diameters ranging from a few to several hundred nanometers [77,81–83]. The most prevailing method for the synthesis of AuNPs is the citrate reduction approach, which was reported by Turkevich *et al.* in 1951 [77]. In a typical synthesis, gold (III) salts are reduced by a reducing agent in aqueous solution. The Au ions undergo nucleation to generate nanoparticles, which are usually in spherical shape due to the thermodynamic causes. To prevent aggregation between AuNPs, a stabilizing agent is required to be adsorbed or chemically bound on the surface of AuNPs. Besides, AuNPs can be prepared by reduction method in organic solvent, though the reducing and stabilizing agents are different to that in aqueous solution [84].

Surface modification provides AuNPs with various properties for further biomedical applications. As was mentioned before; during the particles synthesis a stabilizing agent is required to be bound on the surface of AuNPs to prevent particle aggregation. After synthesis, the stabilizing molecules can be replaced by ligand exchange reaction. Based on strong affinity between thiol moiety and Au surface, thiol-modified ligands are used to bind on the Au surface by formation of Au-sulfur bonds [85]. Thus, the AuNPs can be modified by thiol-based ligands, which contain a thiol group at one end and different functional groups on the other ends. For example, the AuNPs have been modified with citrate [86,87], amine [88–90], peptide [91,92], antibody [93,94], lipid [95,96], and so on.

Although AuNPs have shown biocompatibility *in vitro* [86]; however, few examples indicated that cytotoxicity is related to the surface chemistry of AuNPs [97]. Recently, the synthesis, properties and applications of AuNPs with different surface functionalities were systematically reviewed by Giljohann and coworkers [98]. They speculated that the toxicity of AuNPs was leaded by the surface group itself. For example, for AuNPs modified with cetyltrimethylammonium bromide (CTAB), the cytotoxicity is not exhibited after the excess CTAB is removed by washing [86]. Rotello and coworkers investigated the effects on AuNPs biocompatibility due to chemical functionality and ligand charge. They found amine-functionalized particles were only mildly toxic, and particles functionalized with carboxylic acids were nontoxic under all the condition examined [97]. In addition, the biocompatibility of DNA-AuNPs complexes has been observed [99].

**Figure 7:**
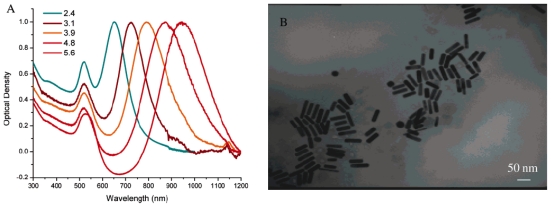
**(A)** Surface plasmon absorption spectra of gold nanorods of different aspect ratios; and **(B)** TEM image of nanorods of aspect ratio of 3.9. Reproduced with permission from [104]^®^ 2006, American Chemical Society.

Recently, the synthesis of non-spherical AuNPs has achieved significant progress [100], especially anisotropic shapes such as nanorods [101]. By controlling the size and shape of AuNPs, the optical characteristics can be significantly changed [102,103]. In the case of spherical AuNPs, the surface plasmon resonance (SPR) absorption is located around 520 nm. For Au nanorods (AuNRs), two plasmon bands are observed (Figure 7) [104]. The band around 520 nm corresponds to the transverse plasmon oscillation (oscillation along the width of AuNR), and the stronger band at longer wavelength corresponds to the longitudinal plasmon oscillation (oscillation along the length of AuNR). The longitudinal plasmon resonance maximum can be shifted into the near-infrared (NIR) region by increasing the aspect ratio of AuNR (*i.e.*, the ratio of length along the long axis to the short axis) [102,104]. In addition, the plasmon band in NIR region can be tuned in silica core-Au shell structure, in which the band wavelength takes red shift with increasing core/shell ration. Red shift of plasmon band helps AuNRs to collect more NIR light, and that is benefic for hyperthermia and photothermal imaging applications.

### 4.2. Therapeutic Applications

**Figure 8:**
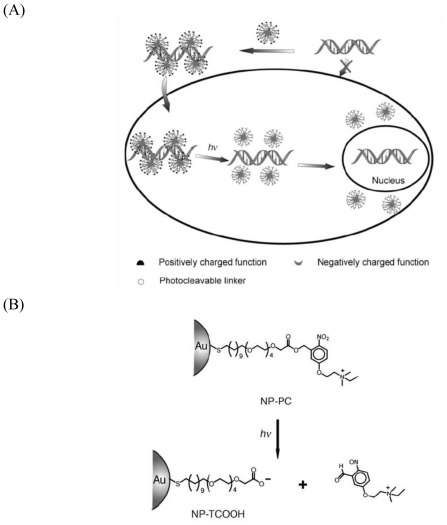
**(A)** Schematic illustration of the release of DNA from the AuNPs modified with photocleable linker; **(B)** The change of electrostatic properties on AuNPs surface before and after UV illumination. Reproduced with permission from [106]^®^ 2006, Wiley-VCH Verlag GmbH & Co. KGaA.

Tunable optical properties and feasible surface modification make AuNPs a promising material for recognition and delivery of therapeutic agents. In general, AuNPs carry molecules via covalent or non-covalent linking. For covalent linking, AuNPs rely on the disulfide linkages to carry drugs, and the utility has already been demonstrated in clinic [105]. For non-covalent linking, electrostatic interaction is an effective strategy to carry DNA, the release of the adsorbed DNA is afforded by changing the electrostatic properties of AuNPs (Figure 8A) [106]. For example, the surface of AuNPs can be modified with a photocleavable *o*-nitrobenzyl ester linker (Figure 8B), which at the end group bears a positively charged quaternary ammonium salt. The positive charge of the material is used to electrostatically interact with DNA. Upon UV irradiation, the nitrobenzyl linkage is cleaved to create an anionic carboxylate group. This resulted in repulsion forces between the nanoparticles and DNA, which afforded the release of the DNA strands.

**Figure 9:**
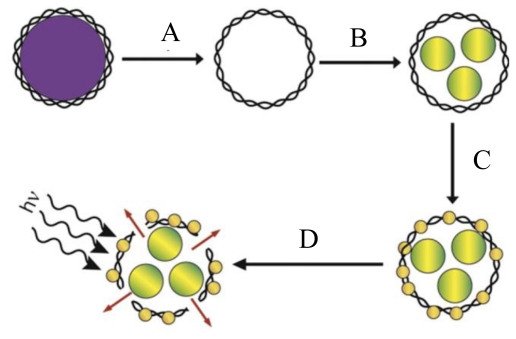
Photothermal effect of gold nanoparticles for releasing encapsulated materials: **(A)** Polyelectrolyte capsule prepared by layer-by-layer process; **(B)** Encapsulation of pharmaceuticals; **(C)** Doping of AuNPs in the shells of capsule; **(D)** Rupture of the shell upon light irradiation. Reproduced with permission from [2]^®^ 2008, Elsevier.

In addition, using the photo-induced heating property of AuNPs, light-responsive delivery systems can be designed (Figure 9) [2,107–109]. Upon NIR irradiation, the heat generated by AuNPs ruptures the outer shell, and the loaded therapeutic agents in the cavity of capsule start to leak through the damaged shell. Skirtach and coworkers have successfully demonstrated that this strategy *in vitro* [110].

Localization of nanoparticles at cancerous tissues can be achieved by two approaches, passive and active targeting methods. In the case of passive targeting, the homing vectors are designed by controlling the size of nanoparticles to leak freely through leaky blood vessel (gaps about 600 nm) [2]. Based on the fact that tumor vasculature is more permeable than that of healthy tissue [111], nanoparticles with an appropriate size can pass through the wall of vessels and be deposited in the surrounding tumorous tissues [112]. On the other hand; in active targeting, the surface of nanoparticles is modified with functional groups such as antibodies or ligands with high affinity for the desired target. This strategy can enhance the specific recognition with cell surface receptors. In addition, to decrease the non-specific binding and potential activation of the reticuloendothelial system (RES), the nanoparticles can be coated by a thiolated poly-ethyleneglycol (PEG) chain [112,113].

Cells and living animals are sensitive to changes in temperature, even rises in a few degrees can produce huge effects. For human beings, body temperature above 37 °C leads to fever and if it exceeds 42 °C may lead to lethality. Based on this fact, a harmless therapy is generated to destroy cancerous tissues based on localized heating, called hyperthermia [102,114]. Adsorption of light by AuNPs can result in hyperthermia. The excited electrons convert light energy into thermal energy and the thermal energy transfer into Au lattices rapidly (≈1 ps) [115,116]. Consequently, the heat from the AuNPs is dissipated into the surrounding environment. Tissue mainly adsorbs visible light and only thin tissues can be penetrated by infrared light. The light penetration depth can be up to a few centimeters in the spectral region 650–900 nm, also known as the biological NIR window [117–119]. To collect the NIR light, the morphology of the AuNPs is designed as either nanorods [104,120,121] or silica core/Au shell structures [117,122]. To obtain the best efficiency, photo-induced heating of AuNPs is recommendable for tumorous tissue localized closer to the skin. For tissue deep in the body, it is favorable to use magnetic particles, which can be heated by applying alternative magnetic hysteresis loop under lower radiofrequency fields.

### 4.3. Diagnostic Imaging Applications

Imaging contrast agents are important for diagnostic techniques in molecular biology and biomedicine, because they can provide strong imaging signals to highlight the specific cells and tissues of interest [117,123]. The first generation of optical contrast agents are based on molecular dyes; for example, rhodamine-6G and malachite green [124]. However, these types of molecular probes suffer from rapid photobleaching. Compared to molecular-based optical contrast agents, AuNPs have many advantages such as insensitivity to photobleaching and tunable optical properties. Due to the phenomenon of SPR, the absorption and scattering cross-section of AuNPs are significantly higher than that of conventional dyes. For example, the molar adsorption coefficient (ɛ) of AuNPs with a diameter of 40 nm is about 7.7 × 10^9^ M^−1^cm^−1^ at a wavelength maximum around 530 nm, and this value is several orders of magnitude higher than that of organic dyes (for example, rodamine-6G has ɛ of 1.2 × 10^5^ M^−1^cm^−1^ at 530 nm) [102].

Combining targeting strategies (passive or active targeting methods), AuNPs can be localized at a specific site and provide excellent imaging quality. As labeling agents, AuNPs have been applied on immunostaining [125–127], single particle tracking [128–130], and X-ray contrasting [131,132]. Besides, AuNPs can be used for imaging the movement of cells adhering to a substrate, this technique is called “phagokinetic tracks” [133–135]. For example; in the case of phagokinetic tracks, cells adhere to the substrate coated with a layer of AuNPs, and then the migration pathway of the cells is obtained by imaging the AuNPs layer with optical transmission microscopy or TEM. The migration of cells does not need to be recorded online. Moreover, many trails can be recorded in parallel on the same substrate and the trails can be imaged *ex situ*, as they consist of areas in the nanoparticles layer that is permanently free of nanoparticles [136].

Traditionally, AuNPs are primarily used for labeling and are observed by TEM due to the high atomic weight of Au atom [137]. Depending on the sizes of AuNPs, different imaging techniques are applied. For instance, large AuNPs (>20 nm) can be imaging using an optical microscope in phase contrast or differential interference contrast (DIC) mode [92], and the detection with an optical microscope only involves scattered light in dark-field microscopy [138]. On the contrary, small AuNPs (<20 nm) only adsorb light to produce heat due to less scattering cross-section, and that can be recorded by photothermal imaging that record local variation of the refractive index by DIC microscopy or by photoacoustic imaging using heat-induced liquid expansion [139]. Other imaging techniques have been already reviewed somewhere else [102,139].

AuNPs display strong native fluorescence and suffer little photobleaching under high excitation power. The fluorescence of AuNPs can be detected by fluorescence spectrometry, fluorescence correlation spectroscopy and fluorescence microscopy. The florescence imaging of AuNPs can reach the single-particle level and has been applied for cell imaging [139]. In addition, AuNPs have been applied using both the fluorescence resonance energy transfer (FRET) technique and quenching effect. In FRET, the emission light and efficiency of energy transfer can be controlled according to the distance between donor and acceptor. Based on FRET, AuNPs can be applied to monitor DNA hybridization and DNA cleavage [140]. In quenching effect, either fluorescent dyes or quantum dots (QDs) can be quenched by AuNPs even if the distances between dyes are much larger than Förster resonance quenching transfer distance [141]. So, an increasing intensity of fluorescence peak is observed as the dyes or QDs and AuNPs are forced apart. Based on this phenomena, Rotello and coworkers designed fluorescence displacement protein sensor array, in which fluorescence was generated by the given protein analytes [142].

## 5. Iron Oxide Nanoparticles (IONPs)

IONPs are a major class of materials with the potential to transform current clinical diagnostic and therapeutic techniques. Due to their unique physical and chemical properties, and ability to function at the cellular and molecular level, IONPs are being actively investigated as an alternative for the development of MRI contrast agents and as carriers for targeted drug delivery. Although the synthesis of IONPs can be dated back several decades, the recent explosion of interest in nanotechnology has significantly expanded the depth of IONPs research. In this section we will explore the extensive range of applications of IONPs in the detection, diagnosis, and treatment of illnesses, such as cancer, cardiovascular disease, and neurological disease. It is envision that IONPs may soon play a significant role in meeting the healthcare needs of tomorrow.

### 5.1. Synthesis of Magnetite (Fe_3_O_4_) Nanoparticles

A lot of approaches have been used to synthesize magnetite, and the synthetic strategies can be categorized in three main methods: (1) two-iron-species, (2) organic-iron-compound, and (3) one-iron-specie. In the case of the two-iron-species method [143–147], magnetite contains two kinds of species of iron ions (*i.e.*, Fe^2+^ and Fe^3+^ ions). The standard protocol is as follow; addition of compound *A* containing Fe^2+^ ion (e.g., FeCl_2_, FeSO_4_, and so on) and compound *B* containing Fe^3+^ ion (e.g., FeCl_3_) into the same deionized water solution while keeping the molar ratio Fe^2^/Fe^3+^ = ½ constant. Then, aqueous ammonia is used to titrate the mixed solution until pH reached 9 or 10 at nitrogen or argon atmosphere. The solution at this step usually turns its color to black after the reaction has been running for a certain time. The black precipitate is magnetite, which can be collected by a magnet. Finally, the magnetite is washed with water until the pH of the supernatant equals to 7.0. Finally; after several washing steps, the magnetite is dried by air. Other modifications such as adding hydrazine [148], and/or bubble air to solution [149] have been published for controlling particle size or the functionality of the as-synthesized magnetite. The second method [150,151], instead of employing inorganic iron complexes, organic-based iron compounds are used as iron source. Although the whole process is more complicated, the magnetite synthesized by this method exhibits more stable magnetic properties and controllable particle size. The standard protocol is briefly described; first the addition of organic iron compounds (e.g., Fe(CO)_5_, Iron pentacarbonyl, *etc.*) into a solution which contains a wide variety of solvents (e.g., benzyl ether, oleic acid, toluene, hexadecane-1,2-diol, *etc.*). After that, the solution is transferred to a Teflon-lined autoclave for 3 to 24 h. The reaction time can be tuned for controlling the particle size of the magnetite. After the solution cools down to room temperature, ethanol is added to facilitate the precipitation of the magnetite. This method is especially useful for synthesizing hydrophobic magnetic nanoparticles. Finally, in the case of the one-iron-species approach [152], only one species of iron compound is used. Moreover, two or more solvents with different polarities are used in this method in order to extract iron-oleic complex in a two-phase solution. For instance, adding FeCl_3_ and sodium oleic acid to water/ethanol/hexane mixed two-phase solution and heating it to 70 °C for several hours to form iron-oleic complex. After that, the iron-oleic complex is separated from the polar solvents by evaporation, and the organic phase is resuspended into 1-octadecene. Then, oleic acid is added followed by heating to 320 °C for 30 min. Finally, ethanol is added to precipitate magnetite nanoparticles. After centrifugation, nanoparticles are washed with hexane and ethanol several times, and then redispersed in hexane or toluene.

### 5.2. Therapeutic Applications

The efficient delivery of therapeutic agents has been a major obstacle in cancer treatment, and has failed almost in 90% of the cases. According to recent investigations, the use of IONPs to efficiently load anticancer drugs can be a promising alternative to overcome this issue. For example, by introducing IONPs into a variety of nanomaterials, the developed magnetite-nanomaterial composite not only can overcome the problem of the drug resistance but also open other possibilities for cancer treatment. The IONP-based therapeutic applications can be categorized into three major fields: pH-and temperature-sensitive drug release, and hyperthermia. In the case of pH-sensitive drug release [151,153,154], the IONPs are employed as core and silica is used to cover the surface of the nanoparticles as shell to form a IONP/SiO_2_ (core/shell) nanocomposite. The silica surface can be further functionalized with amine groups to conjugate drugs bearing a carboxylic group by forming an amide bond [145,155]. Other methods using electrostatic interaction between the therapeutic agent and the nanocomposite have also been reported [156,157]. Either by chemical bonding or electrostatic interaction, the drug can be released in the acidic conditions (pH value is around 4–5) by breaking these interactions. Because cancer cells and around of tumor tissues show a relative acidic environment, the agent will be released efficiently when the drug-loaded magnetite/SiO_2_ nanocomposite reaches to the desired site [158]. On the other hand, temperature-sensitive delivery systems is a novel concept for drug release, which involves the use of magnetic nanoparticles as triggers for the release of guest molecules [159]. First, surface functionalized IONPs are used as core, and a layer of temperature-sensitive polymers is coated onto the surface of the particles. Such IONP/polymer material exhibits a great potential for temperature-responsive controlled drug release platforms. By applying alternating magnetic field to the nanocomposite, the IONPs in the core generate heat, which induces the polymer shell to release the payload. The other major area for therapeutic applications of IONPs is hyperthermia [146,160,161]. In this therapeutic approach the body tissue is exposed to high temperatures to either eradicate cancer cells or to make them more sensitive to other treatments, such as radiation and anti-cancer drugs. When applying an alternative external magnetic field, the IONPs contained in the drug carrier absorb electromagnetic waves and release heat. Moreover, when the magnetite-containing nanocomposite is loaded with anti-cancer agents; this material can release the therapeutic molecules and generate heat simultaneously at the desired site.

### 5.3. Diagnostic Imaging Applications

IONPs have been widely used in *in vivo* bio-imaging applications, due to their biocompatibility and paramagnetic properties. MRI is one of the most used imaging techniques in clinic. A major advantage of MRI is that it can be used to perform real-time imaging of dynamic biodistribution and clearance of magnetic nanoparticles [162]. On the other hand, IONPs can also be applied into fluorescence imaging. The imaging applications of magnetite can be categorized into two main fields: MRI and fluorescence imaging. It has been demonstrated that by using magnetite-based nanomaterials, the MR imaging contrast enhancement can be improved [148,163–165]. In addition, their biocompatibility and high relaxivity (r_2_) compared with commercial MRI contrast agents, makes this material a promising MRI contrast agent. Furthermore, one can envision that the IONPs can be loaded with therapeutic agents by building a porous structure around the surface of the magnetite, core/shell approach. When the drug-loaded IONPs are uptaken by cancer cells; the particles would perform as theranostic platform by simultaneously releasing the drug molecules and imaging the intracellular environment. In addition, IONPs can work as multimodal probes with the addition of fluorophores [143,144,152]. For instance, IONPs can act as a core, and silica shell can be coated on the surface of the magnetite core. Then, fluorophore molecules are conjugated on the surface of the silica shell. The remaining functional groups can be used for further with biomolecules, enabling the applications of the nanocomposites for specific recognition of biological targets.

Finally, theranostic applications of IONPs can be envisioned by combining the multimodal and therapeutic properties of this platform [150]. Briefly, the IONP-SiO_2_ core-shell structure will be chemically modified with a chromophore molecule to form a multi-functionalized nanocomposite. The magnetite core provides the imaging modality of MRI, and the fluorophore is suitable for fluorescence labeling after irradiation with visible light or infrared. At the same time, the compound can be a photosensitizer, which will generate free radicals or peroxides to induce the apoptosis of cancer cells.

## 6. Quantum Dots (QDs)

**Figure 10:**
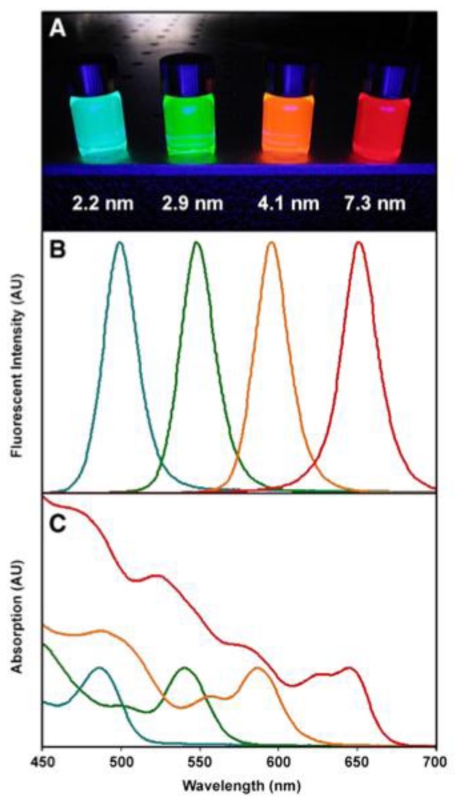
**(A)** The emitting light of QDs is size-tunable; **(B)** Their emission spectrum is usually narrow; **(C)** The broad adsorption spectrum of QDs. Reproduced with permission from [169]^®^ 2008, Elsevier.

Quantum dots (QDs) have been an attractive research field for almost 30 years because of their unique optical properties. They are semi-conductive nanocrystals composed by group I–VII, II–VI or III–V [166]. Unlike the bulk semi-conductive crystals, QDs exhibit novel features such as size-tunable optical properties (Figure 10A), broad absorption (Figure 10B) and narrow emission bands (Figure 10C), large two-photon absorption cross section, and long observation time compared to the conventional fluorescence dyes [167–169]. On the last three decades, QDs have been successfully characterized and applied to different areas such as cellular imaging, *in vivo* and *in vitro* tumor targeting, and other biomedical applications [170–173].

### 6.1. Synthesis

The synthesis of QDs has been developing for more than 30 years. In the 1980s, the first strategies for synthesizing QDs were designed in liquid-phase systems [174]. However, several disadvantages such as low fluorescence efficiency, broad size distribution, and poor crystal packing limited the possibility in optical applications. Since then, several synthetic approaches have been developed and investigated. The main improvements include the finding of synthetic methods in oil-phase, the core/shell structured or surface-modified QDs, the substitution of highly-toxic and/or cost precursors. Others studies have also shown that the reagents, amount of starting material, and temperature all affect the quality of QDs such as the morphology and optical properties (*i.e.*, quantum efficiency) [175,176].

In 1993, Bawendi and coworkers introduced a new strategy based on the pyrolysis of organometallic reagents by injection into a hot coordinating solvent [177]. The solvent is trioctylphosphine oxide (TOPO) which is placed in the reaction vessel and heated to 300 °C. The cadmium precursor is dimethylcadmium (Cd(CH_3_)_2_), which is handled in airless condition. Trioctylphosphine (TOP) then acts as the solvent for the Cd and Se precursors. Two solutions, Cd(CH_3_)_2_/TOP and TOPSe/TOP, are prepared, combined and loaded into a syringe. The heating source is removed from the reaction vessel and the solution in the syringe is rapidly injected into the vessel with rapid stirring. The injection of the reagent solution at room temperature causes a sudden decrease in temperature to 180 °C. Heating is resumed and the temperature is gradually raised from 230 to 300 °C. Aliquots of the reaction solution are collected from vessel at intervals of 5 to 10 min and absorption spectra are recorded to monitor the growth of the nanocrystals. The quality of samples is determined by the width in the absorption spectra, which is related to the size and distribution of QD nanocrystals. To isolate the product, methanol is added to flocculate. The flocculent is then separated by centrifugation, dispersed in 1-butanol and further centrifuged again to yield a solution of QD nanocrystals and a precipitate containing the by-products. Additional amount of methanol is added to the clear solution to produce a flocculent. Finally, the flocculent is rinsed in methanol and dried under high vacuum. The final products are TOP/TOPO capped QD nanocrystals. The as-synthesized QDs are monodisperse due to the separation of the nucleation and growth steps during synthesis. Although this method overcomes some of the issues with broad size distribution, the quantum yield is still quite low (*i.e.*, ~10%). Moreover, this synthetic strategy uses Cd(CH_3_)_2_ as the precursor of Cd, which is believed to have high toxicity *in vivo*. In addition, this synthetic approach is high-cost and must be treated in airless and water-excluded condition, which causes difficulties for practical applications.

As was mentioned above, the toxicity of QDs is a severe problem for *in vivo* applications. To overcome that issue, researchers have developed core/shell approaches to passivate the QD surface. The core/shell QD nanostructure; first reported by Hines and coworkers [178], is a successful approach to develop biocompatible nanoparticles. They synthesized ZnS-capped CdSe nanocrystals, increasing the quantum yield up to 50%. The methodology is similar to the method described above; but with two additional solutions, bis(trimethylsilyl)sulfide [(TMS)_2_S] and dimethylzinc [(CH_3_)_2_S]. After the addition of Cd/Se/TOP stock solutions, the Zn/S/TOP solution is injected at an appropriate temperature. Bawendi and coworkers also synthesized core/shell nanostructures of CdSe/ZnS [167]. They use different reagents, *i.e.*, Zn(C_2_H_5_)_2_ and (TMS)_2_S as the Zn and S precursors, respectively. The key point for the high yield of this approach is because ZnS shell eliminates the unsaturated bonds on the crystal surface and thus increases the monodispersity. Although the quantum yield increases, the lattice mismatch between the CdSe core and ZnS shell is large and thus this core/shell nanostructured QDs form surface defects when the ZnS layer is thicker than 2 atoms. The lattice mismatch of some shell materials such as CdS and ZnSe are lower, but the deference of band gap between core and shell materials result in poor stability of QDs [179].

Due to the high cost and environmental issues the current synthetic methods for QDs are not ideal approaches for industrial production. To make the synthesis more friendly to the environment, Peng and coworkers replaced the Cd(CH_3_)_2_ reagent by CdO [180]. In this one pot approach, CdO, TOPO and either hexylphosphonic acid (HPA) or tetradecylphosphonic acid (TDPA) are loaded in the reaction vessel. Once the solution becomes clear and colorless at 270 °C, Se in tri-n- butylphosphine (TBP/Se) is then injected in to vessel to initiate the formation of QD nanocrystals. The authors showed that these QDs can be synthesized in high yield and quality, and this methodology can be applied for the synthesis of CdS, CdSe and CdTe QD nanocrystals. Moreover, Weller and coworkers developed another synthetic methodology using Cd(CH_3_CO_2_)_2_ as precursor [181]. In this method, a stock TOP/Se is added to a mixture of TOPO/HAD/TDPA at 120 °C and is then heated to 300 °C. The TOP/Cd(CH_3_CO_2_)_2_ solution is added to TOPO/HAD/TDPA solution. The “green” synthetic strategies discussed above reach reasonable quantum yield (*i.e.*, >50%). To enhance the quantum yield, Mekis and coworkers coated CdS shell on the CdSe, which was synthesized by adding H_2_S gas into the CdSe solution at 140 °C [182]. With this method the quantum yield can be raised to 80 to 90%. An additional strategy to synthesize QDs through green chemistry is by the replacement of TOPO [183]. Peng and coworkers found that vegetable oil (oleic acid) could be used as the reagent to complex Cd from CdO. This method replaced TOPO with the non-coordinating solvent (*i.e.*, octadecene). The utilization of non-coordinating solvents further enhances the possibility of implementing green chemistry into the design of synthetic schemes for colloidal QD nanocrystals.

### 6.2. Therapeutic Applications

The major concern for the application of QDs toward biomedicine is the high toxicity resulted from the presence of heavy metals such as Cd, Hg, *etc.* in the nanoparticles [184]. These elements can be potent toxins, neurotoxins, and/or teratogens depending on the dosage, complexation, and accumulation in the liver and nervous system. Therefore, the QD/SiO_2_ (core/shell) approach has been a potential alternative to reduce the ion release from metal-containing core, consequently more biocompatible materials [185]. QDs feature versatile surface chemistry that allows the attachment of ligands and loading of both hydrophilic and hydrophobic therapeutic agents [1]. However, being a relatively novel technology, QD-based drug delivery systems are not as robust and well-established as other liposome- or polymer-based platforms. Therefore, a more common approach is the utilization of QDs as fluorescent markers for tagging conventional drug carriers. For visualization of these vehicles, QDs have been either linked to the surface or incorporated inside of the liposomes [186,187]. In addition, further functionalization of QDs with peptides and/or antibody can be another strategy for both *in vitro* and *in vivo* targeting [188]. Future works in this area will focus on the incorporation of environmentally responsive materials for controlled drug release; non-fouling surface coatings for improved biodistribution; and multistage targeting functionality for enhanced therapeutic specificity.

### 6.3. Diagnostic Imaging Applications

QDs exhibit several advantages for biological imaging such as high quantum yields, high molar extinction coefficients (1–2 orders of magnitude higher than traditional organic dyes), strong resistance to photobleaching and chemical degradation, long fluorescence lifetimes (>10 ns), broad excitation spectra but narrow emission spectra (20–30 nm full width at half maximum), and large effective Stokes shifts. Despite these novel optical features, QDs do not perform optimally for *in vivo* imaging applications. The emitting light in the visible regime cannot penetrate through the skin of animal subjects and thus triggers the development of QDs emitting wavelengths at NIR region [189]. In addition, a number of studies have reported QD flickering in cellular specimens, a phenomenon termed “blinking” [190–192]. This issue can be overcome by passivation of the QD surface with thiol moieties or by using QDs in suspension.

Numerous cellular components and proteins have been labeled and visualized with functionalized QDs, such as the nuclei, mitochondria, microtubules, actin filaments, cytokeratin, endocytic compartments, mortalin, and chaperonin proteins [171,193,194]. The high sensitivity of QDs combined with a wide number of well-separated colors all excitable by a single light source makes these nanoprobes ideal for multiplexed cellular imaging [195]. As a result of their high photo-stability, QDs can be effectively tracked over an extended period of time in order to monitor cellular dynamics including movement, differentiation, and final fate [196–199]. In addition, other techniques have been implemented to QDs. For example, FRET and QD have been applied for imaging since 1996 [200]. Conjugated with the DNA sequence or other bio-macro molecules, we can track the reaction dynamics by observing the change in light strength. FRET has been used in immunological analysis, nucleic acid targeting and biological molecule interaction and conformation change. Others *in vitro* applications including the tracking of RNA interference [201], targeting surface proteins in living cells [202], detection of bacteria [203], and coupling with other nanoparticles such as carbon nanotubes [204]. Additionally, QD “peptide toolkit” has been constructed for the creation of small, buffer soluble, mono-disperse peptide-coated QDs with high colloidal stability [205]. QD-based probes have been used for co-immunoprecipitation and Western blot analysis, allowing for simpler and faster image acquisition and quantification than traditional methods [206–208].

One of the main goals of QDs research is to eventually translate their imaging applications toward clinic. Significant improvements in QD synthesis, surface functionalization, and conjugation techniques combined with their photo-stability and brightness have made QDs invaluable tools for *in vivo* imaging. QDs that emit in the NIR region are suitable for biomedical applications because of low tissue absorption, scattering, and auto-fluorescence in this region, which leads to high photon penetration in tissues [209]. QDs also have been used as cell markers to study extravasation in small animal models. QD-labeled tumor cells were intravenously injected into live mice and there were no distinguishable differences in behavior between the QD-labeled tumor cells and unlabeled cells [210]. Using multi-photon microscopy, QDs can differentiate tumor vessels from perivascular cells and matrix better than traditional fluorescence-labeled dextran vessel markers [211]. Moreover, visualization of blood vessels in the chick chorioallantoic membrane, a popular model for studying various aspects of blood vessel development such as angiogenesis, was recently achieved with QDs imaging [212].

*In vivo* targeting and imaging is very challenging due to the relatively large size compared to molecular agents and short blood circulation time. The first report to demonstrate *in vivo* targeting of QD conjugates employed peptides as the targeting ligands [213]. QD materials for *in vivo* imaging can be synthesized by either surfactant exchange or by insulation of the original hydrophobic QDs within a heterofunctional amphiphilic coating [195,214]. To further functionalize the QD particles different strategies have been used such as ligand exchange with small thiol-containing molecules [215,216] (*i.e.*, oligomeric phosphines or dendrons or peptides); encapsulation by a layer of amphiphilic diblock [217] or triblock copolymers [218]; silica coating [219], phospholipid micelles [196], polymer beads [220], polymer shells [221], or amphiphilic polysaccharides [222]; and combination of layers of different molecules conferring the required colloidal stability to QDs [223,224]. Once the QDs have been functionalized with hydrophilic ligands, those can serve as anchoring points for the chemical attachment of biomolecules to functionalize the QDs surface. QDs ligands containing either an amine or a carboxyl group offer the possibility to crosslink molecules containing a thiol group [196,213,225] or an *N*-hydroxysuccinimyl-ester moiety [205,226] using standard bioconjugation reactions.

One of the most promising applications for QDs is the development of multimodal nanoprobes for *in vitro* and *in vivo* imaging. A series of core/shell CdSe/Zn_1−x_Mn_x_S nanoparticles have been synthesized for use in both optical imaging and MRI [227]. Bi-functional nanocomposite systems consisting of Fe_2_O_3_ magnetic nanoparticles and CdSe QDs have been synthesized [228]. QDs can be coated with paramagnetic and PEG lipid derivatives for use as detectable and targeted probes with MRI [229]. These QDs are useful as dual-modality contrast agents due to their high relaxivity and ability to retain their optical properties. Several other QD-based probes for both fluorescence imaging and MRI have also been reported [229–231]. A multi-purpose material that consists of a polymerfunctionalized Fe_2_O_3_ coated with a CdSe-ZnS shell and further modified with antibodies have been used to magnetically capture breast cancer cells and view them with fluorescence imaging [232]. In addition, magnetic QDs composed of CdS-FePt have also been synthesized [233].

With the development of smaller non-Cd based multifunctional QDs and further improvement on conjugation strategy, it is expected that QDs will achieve optimal tumor targeting efficacy with acceptable toxicity profile for clinical application in the near future using either by near-infrared fluorescence (NIRF) imaging or through multimodal imaging.

## 7. Conclusions

Inorganic-organic hybrid nanomaterials have been synthesized by a wide variety of synthetic strategies. The inclusion of organic moieties both at the molecular and macromolecular level allows these particles to be modified for an extensive diversity of biomedical applications. The ultimate goal in creating these multifunctional platforms is the efficient and specific treatment as well as the diagnosis of diseases. Some key drawbacks need to be overcome before these hybrid nanomaterials can be used in clinics, such as the biocompatibility, *in vivo* targeting efficacy, and long term stability. We envision that the continuous development of novel inorganic-organic hybrid nanomaterials and their application in living organisms will open the way to new avenues of diagnosis and therapy with potential applications in the clinic.
